# Activity impairment, health-related quality of life, productivity, and self-reported resource use and associated costs of uncomplicated urinary tract infection among women in the United States

**DOI:** 10.1371/journal.pone.0277728

**Published:** 2023-02-01

**Authors:** Jeffrey Thompson, Alen Marijam, Fanny S. Mitrani-Gold, Jonathon Wright, Ashish V. Joshi

**Affiliations:** 1 Cerner Enviza, Malvern, Pennsylvania, United States of America; 2 GSK, Collegeville, Pennsylvania, United States of America; Johns Hopkins Medicine, UNITED STATES

## Abstract

**Background:**

Uncomplicated urinary tract infections (uUTIs) are among the most common infections in the US. Only a few studies, however, describe the impact of uUTIs from the patient perspective.

**Methods:**

A cross-sectional online survey of US women aged ≥18 years was performed assessing uUTI burden regarding activity impairment, health-related quality of life (HRQoL), workplace productivity, healthcare resource use (HRU), and costs. Participants who self-reported a uUTI in the prior 60 days treated with ≥1 oral antibiotic were included. Activity impairment was assessed with the Activity Impairment Assessment scale. HRQoL was assessed using a modified Short Form 36 (SF-36). Direct costs were sum of out-of-pocket expenditures and monetized HRU; indirect costs were calculated using Work Productivity and Activity Impairment (WPAI). Participants were stratified by uUTI recurrence, number of prescribed antibiotics for recent uUTI and therapy appropriateness (1 first-line/1 second-line/multiple antibiotics). Multivariable regression analysis assessed the relationship between stratifications and outcomes while controlling for demographic/clinical characteristics. Propensity score matching was used to compare participants to a matched population from the 2020 National Health and Wellness Survey (NHWS), to control for any impact of COVID-19 on responses.

**Results:**

Among 375 participants, impaired activities included sexual intercourse (66.9%), sleep (60.8%) and exercise (52.3%). HRQoL was worse (p<0.0001) than the NHWS population (46.4 vs. 51.3 [physical component score]; 40.0 vs. 46.9 [mental component score]; 0.63 vs. 0.72 [health utility index]). All included WPAI assessments were worse for uUTI cohort vs. NHWS (p<0.0001). Adjusted direct costs were higher for participants receiving 2 vs. 1 antibiotic ($2090 vs. $776; p<0.0001) and receiving multiple antibiotics vs. 1 first-line ($1642 vs. $875; p = 0.002). Recurrent uUTI was associated with increased activity impairment, worse HRQoL, and costs vs. non-recurrent.

**Conclusions:**

uUTIs were associated with increased activity impairment, worse productivity, and reduced HRQoL. Higher costs were found vs. a matched population.

## Introduction

Symptomatic uncomplicated urinary tract infections (uUTI) are one of the most common infections in the United States (US) [1]. They are defined by the presence of dysuria, urinary frequency, urgency, and suprapubic pain in women lacking fever and functional or anatomical abnormalities of the urinary tract, with no recent urinary instrumentation [2]. Urinary tract infections (UTIs) account for a substantial proportion of antibiotics prescribed in primary care [3], and account for 10.5 million ambulatory care visits due to UTI or cystitis in the US, or 0.9% of all ambulatory care visits [4]. The majority of uUTIs are caused by *Escherichia coli* (*E*. *coli*) [1] and standard of care for uUTIs is empiric oral antimicrobial agents with activity against certain Gram-positive and Gram-negative bacteria, including: fosfomycin, nitrofurantoin, trimethoprim-sulfamethoxazole, and β-lactams (amoxicillin-clavulanate, cefdinir, cefaclor, cefpodoxime-proxetil, and cephalexin) as alternate agents in patients with allergies/intolerance to first line [5]. There is, however, increasing antimicrobial resistance among *E*. *coli*, which is a global problem [6].

UTIs are common infections, occurring in an estimated 1 in 3 women by the age of 24 years, or 50–60% of all women in a lifetime [1]. UTIs have a substantial effect on quality of life and represent a considerable healthcare burden [7]. For example, in a population survey conducted in England in 2014, Butler et al. [3] found that 15% of patients reported UTIs impacting their daily lives “a great deal”, 37% reported it affecting their daily lives “a fair amount”, and 95% reported contacting a healthcare professional about their most recent UTI [3]. Furthermore, Ellis and Verma [8] found that quality of life scores (based on the Short-Form 36 questionnaire) were lower across all domains of the assessment for US women with outpatient UTI compared with healthy controls [8]. The evaluation and treatment of UTI costs several billion dollars per year globally, and approximately $2 billion per year in the US [9].

While the effects of UTI on quality of life have been investigated previously [7,8], contemporary studies regarding the key drivers of health-related quality of life (HRQoL), work productivity loss, healthcare resources use, direct and indirect costs, activity impairment, and treatment satisfaction are lacking. Furthermore, earlier studies on UTI and have not made a clear distinction between complicated UTI and uUTI. Thus, in order to better understand these factors, we conducted a survey examining patient-reported activity impairment, HRQoL, workplace productivity, uUTI-related healthcare resource use (HRU), and costs in US women with a self-reported uUTI in the past 60 days that was treated with an oral antibiotic.

## Materials and methods

### Study design

This was a cross-sectional survey of women who self-reported a UTI episode in the past 60 days that was treated with an oral antibiotic. Women who had previously agreed to participate in general population surveys conducted by Dynata, EMI, Lucid/Federated or Kantar Profiles, were invited via email to participate. Details of the source populations are provided in S1 Table in S1 File. After online screening of inclusion/exclusion criteria, eligible participants (i.e., those with a uUTI within the study recall period) provided informed consent prior to completing an online questionnaire regarding their most recent uUTI. Respondents were permitted to complete the online survey only once and were compensated for their participation time.

### Participants

Women were eligible to participate in the survey if they were aged ≥ 12 years (although no respondents were younger than 18 years), living in the US, able to read English, and reported a UTI in the 60 days prior to participation for which they received oral antibiotic therapy. In order to ensure that the inclusion UTI episode was uncomplicated, participants were excluded if they self-reported a diagnosis of any of the following, indicative of complicated UTI (cUTI), in the 6-month period before oral antibiotic treatment for the inclusion episode: urologic abnormalities, ureteral abnormalities, interstitial cystitis, pyelonephritis, kidney stones, renal failure, congenital urological abnormalities, organ transplant, or neurological disease. Additional exclusion criteria were the following: self-reported diabetes with an unknown or uncontrolled glycosylated hemoglobin level (≥ 7%); self-reported receipt of any immunosuppressive therapy at the time of developing a UTI; any UTI identified in the previous 60 days as having occurred during an inpatient hospitalization or stay at a long-term care facility; antibiotic treatment received for inclusion UTI during an inpatient hospitalization; pregnancy at the time of recent uUTI; asymptomatic bacteriuria (i.e., positive urine culture with no UTI symptoms present); and diagnosis of COVID-19 in the previous 12 months.

### Study data

Data collected during the survey included demographics (age, insurance type, marital status, race/ethnicity, employment type, education, type of residence [rural/urban], US region, household income); health characteristics (i.e., height, weight, body mass index, smoking status, alcohol use); comorbidities; healthcare resource utilization; UTI history (with ≥ 2 self-reported UTIs in the previous 6 months or ≥ 3 uUTIs in the past year considered recurrent); symptom severity; activities impacted by inclusion uUTI; treatments used for recent uUTI and any prior UTI (including antibiotics used); HRU; and self-reported direct costs.

### Objectives

The primary objective of the study was to assess activity impairment associated with uUTI. Secondary endpoints were assessment of HRQoL, workplace productivity, HRU and associated costs (direct and indirect) associated with the participants’ most recent uUTI event. Treatment satisfaction with the most recent oral antibiotic received was examined as an exploratory endpoint (See Supplementary methods).

The Activity Impairment Assessment (AIA) was used to measure the impact of uUTI on daily activities. The AIA is a validated 5-item self-administered questionnaire assessing the amount of time that daily activities have been impaired by uUTI symptoms [10]. Responses are given on a 5-point Likert-type scale from 0–4 (“none of the time”–“all of the time”). AIA scores range from 0 to 20 with higher scores denoting higher impairment. HRQoL was assessed using a modified version of the Short Form 36 version 2 (SF-36v2) with a recall period adjusted to the time of the most recent uUTI. The SF-36v2 is a 36-item self-administered questionnaire covering measures of 8 health domains [11]. We utilized the Physical Component Score (PCS), Mental Component Score (MCS) and health utility index (SF-6D) from this questionnaire. HRU was measured as the number of self-reported healthcare visits utilized to treat the inclusion uUTI, encompassing: primary care provider (PCP) office visits, specialist (obstetrician/gynecologist) office visits, urgent care visits, emergency room (ER) visits, and hospitalizations. Direct costs were calculated as the sum of self-reported out-of-pocket costs (including payments for doctor(s) visits, prescription medications, over-the-counter treatments, costs associated with travel to receive treatment, childcare costs, etc.) and HRU monetized with Medical Expenditure Panel Survey estimates [12]. Indirect costs were calculated via Work Productivity and Activity Impairment (WPAI) metrics [13] monetized with Bureau of Labor Statistics estimates [14].

### Statistical analyses

Outcomes were reported with descriptive statistics, chi-squared tests, and t-tests. Participants were stratified by recurrent uUTI (yes/no); the number of oral antibiotics prescribed for their most recent uUTI (1, 2 or ≥ 3); and by appropriateness of therapy, defined as 1 appropriate (first-line) antibiotic only, 1 inappropriate (not first-line) antibiotic only, or multiple antibiotics (any line) per Infectious Diseases Society of America guidelines [5]. Multivariate regression with generalized linear models was used to compare strata for the following outcomes of interest: activity impairment, HRQoL, total uUTI-related costs (direct and out-of-pocket), WPAI measures (absenteeism, presenteeism, overall work impairment, impact on daily activities). Covariates were chosen via a stepwise selection process using SAS PROC HPGENSELECT (SAS Institute Inc., NC, USA) on the full cohort sample. For number of therapies, results were adjusted for age, race/ethnicity, employment, and use of any second-line antibiotic. For appropriateness of therapy, adjustments were made for age, race/ethnicity, employment, region, and any physician-ordered urine testing.

PCS and MCS were compared to the general US population average (Optum Inc., MN, USA) and categorized as “Same or Better” than the general population, “Below” the general population or “Well below” the general population, defined as scores lower by ≥ 5 points (the minimum clinically important difference for PCS and MCS) [15].

Propensity score matching (1:1) was used to assess uUTI burden in the study cohort compared with a matched US population from the National Health and Wellness Survey (NHWS) 2020. The NHWS is a self-administered, internet-based survey of a nationwide sample of adults (aged ≥ 18 years) stratified by age, gender, and racial/ethnic groups to represent the demographic composition of the US adult population [16]. This analysis was performed to address the potential impact that the COVID-19 pandemic may have had on study measure responses (such as activities involving social contact) and was used to assess the incremental burden associated with uUTI when compared to a matched population that were also survey participants during a similar time period in 2020.

This study was originally scoped to include 850 participants meeting all eligibility criteria. The final study sample used for analysis included 375 respondents. Precision analysis estimates for descriptive statistics show that at a 95% confidence interval (CI) the associated margin of error (MOE) for the minimum sample size of 385 is ±5%. At a 99% CI the associated MOE for a sample size of 339 is ±7%. Power calculations for linear multiple regression modelling (including composite AIA score, HRQoL MCS/PCS scores, etc.) indicate that a sample size of 160 was required to reach a power of 80% (α = 0.05) for an effect size of 5%. The current study sample size with a minimum of 375 is powered at 99% (α = 0.05) to detect a 5% effect size when performing linear multiple regression modelling.

### Ethics

Ethical approval of the study protocol was provided by Pearl IRB LLC (Indianapolis, USA; reference #20-KANT-222). Participants provided written informed consent (via an online consent form) prior to their involvement in the study. No personally identifiable information was collected as part of the study. The study complied with all applicable privacy laws.

## Results

### Participants

In total, the questionnaire was accessed by 54,020 individuals and 375 eligible participants completed the questionnaire between July 28 and September 28, 2020. Of these, 43.5% were categorized as having recurrent uUTI based on their self-reported uUTI history. Additionally, 56.8% of participants reported using ≥ 1 first-line oral antibiotic (trimethoprim-sulfamethoxazole [TMP-SMX], nitrofurantoin [NFT], fosfomycin) and 50.9% reported using ≥ 1 second-line (ciprofloxacin, ofloxacin, levofloxacin) or alternate antibiotic (amoxicillin-clavulanate, cefdinir, cefaclor, cefpodoxime-proxetil, cephalexin) to treat their most recent uUTI. Across all participants, 62.7%, 23.5%, and 13.9% reported having used 1, 2 or ≥ 3 oral antibiotics to treat their most recent uUTI, respectively. The antibiotic most commonly used to treat participants’ most recent uUTI was TMP-SMX (38.7%) followed by ciprofloxacin (22.7%) and NFT (18.9%). Most participants (82.7%) received some type of urine test, with 63.5% receiving a urine culture test, and 13.1% receiving an antimicrobial susceptibility test. Following physician-ordered urine testing, 19.5% had their oral antibiotic changed.

Participant demographic data and clinical characteristics are shown in Table 1. Study participants were evenly distributed across all age groups; the majority were white (84.8%), 44.0% were married, 34.1% were in full-time employment, and 41.1% had employer-provided health insurance. Many participants were never smokers (45.9%), and the most common current comorbidities were depression (36.5%), anxiety (32.3%), and hypertension (19.7%). Significant differences in demographic data and clinical characteristics were observed between stratification cohorts (S2 and S3 Tables in S1 File). Participants in rural areas were more likely to report using 2 antibiotics than ≥3 (31.8% vs. 15.4%; p = 0.018) and participants in urban areas were more likely to report using ≥3 antibiotics than 2 (38.5% vs. 19.3%; p = 0.018; S2 Table in S1 File). Furthermore, those who received 2 antibiotics were more likely to have current comorbid irritable bowel syndrome (IBS) than those who received 1 antibiotic (18.2% vs. 8.1%; p = 0.009) and were less likely to report current comorbid anxiety than those who received 3 antibiotics (29.5% vs. 50.0%; p = 0.016; S2 Table in S1 File). Participants who received 1 appropriate antibiotic only were more likely to have employer-provided insurance than those who received 1 inappropriate antibiotic only (48.8% vs. 39.3%; p < 0.05; S3 Table in S1 File). Participants in the multiple antibiotics cohort were more likely to have Medicare than the 1 inappropriate antibiotic only cohort (29.3% vs. 23.2%; p < 0.05), and were more likely to be from the Mid-West (54.3% vs. 34.1%; p = 0.001), have a household income of $50,000–74,999 (27.1% vs. 14.6%; p = 0.029), and be currently experiencing IBS than the 1 appropriate antibiotic only cohort (15.7% vs. 6.5%; p = 0.019; S3 Table in S1 File). The mean number of all-cause ER visits in the past 12 months was higher in the multiple antibiotics cohort than in the 1 appropriate antibiotic only cohort (0.7 vs. 0.4; p = 0.015), but a greater proportion of the appropriate antibiotic cohort reported an all-cause urgent care visit in the past 12 months than the inappropriate antibiotic cohort (40.7% vs. 27.7%; p = 0.037; S3 Table in S1 File).

**Table 1:** Participant demographics and clinical characteristics.

Participants with uUTI (n = 375)	
**Age, years (n [%])**	
18–29	66 (17.6)
30–39	95 (25.3)
40–49	76 (20.3)
50–64	85 (22.7)
65+	53 (14.1)
**Insurance type (n [%])**	
Employer provided	154 (41.1)
State health exchange (’Obamacare’)	26 (6.9)
Dual	12 (3.2)
Medicare	81 (21.6)
Medicaid	62 (16.5)
No insurance	21 (5.6)
Other	19 (5.1)
**Marital status (n [%])**	
Married	165 (44.0)
Single, never married	73 (19.5)
Divorced	63 (16.8)
Separated	9 (2.4)
Widowed	19 (5.1)
Living with partner	44 (11.7)
Other/Not reported	2 (0.5)
**Race/ethnicity (n [%])**	
African American/Black	13 (3.5)
Asian or Pacific Islander	11 (2.9)
Hispanic^a^	22 (5.9)
White	318 (84.8)
Multiple/Other	11 (2.9)
**Employment type (n [%])**	
Full-time	128 (34.1)
Part-time	38 (10.1)
Self-employed	12 (3.2)
Homemaker	51 (13.6)
Retired	58 (15.5)
Unemployed	50 (13.3)
Disabled	27 (7.2)
Student	8 (2.1)
[Decline to answer]	3 (0.8)
**Education (n [%])**	
Less than high school	3 (0.8)
Completed some high school	13 (3.5)
High school graduate or equivalent	55 (14.7)
Completed some college, but no degree	106 (28.3)
Associate’s degree	59 (15.7)
College graduate	91 (24.3)
Completed some graduate school, but no degree	13 (3.5)
Completed graduate school	34 (9.1)
[Decline to answer]	1 (0.3)
**Residence (n [%])**	
Urban/City	88 (23.5)
Suburban	196 (52.3)
Rural	91 (24.3)
**US region (n [%])**	
Northeast	75 (20.0)
South	75 (20.0)
Midwest	166 (44.3)
West	59 (15.7)
**Household income (n [%])**	
Less than $15,000	37 (9.9)
$15,000 to $24,999	31 (8.3)
$25,000 to $34,999	33 (8.8)
$35,000 to $49,999	47 (12.5)
$50,000 to $74,999	80 (21.3)
$75,000 to $99,999	60 (16.0)
$100,000 to $124,999	35 (9.3)
$125,000 to $149,999	20 (5.3)
$150,000+	26 (6.9)
[Decline to answer]	6 (1.6)
**Smoking status (n [%])**	
Never	172 (45.9)
Used To	88 (23.5)
Once a month or less	11 (2.9)
2–3 times per month	6 (1.6)
Once a week	3 (0.8)
2–3 times per week	5 (1.3)
4–6 times per week	11 (2.9)
Daily	79 (21.1)
**Alcohol use (n [%])**	
Never	40 (10.7)
Used To	69 (18.4)
Once a month or less	88 (23.5)
2–3 times per month	53 (14.1)
Once a week	54 (14.4)
2–3 times per week	42 (11.2)
4–6 times per week	14 (3.7)
Daily	15 (4.0)
**Comorbidities, current (n [%])**	
AIDS/HIV	2 (0.5)
Asthma	69 (18.4)
Cancer	21 (5.6)
Cerebrovascular accident or transient ischemic attack	5 (1.3)
Chronic anxiety	121 (32.3)
Chronic pulmonary disease	30 (8.0)
Congestive heart failure	4 (1.1)
Connective tissue disease	8 (2.1)
Dementia	1 (0.3)
Depression	137 (36.5)
Diabetes (type 1)	2 (0.5)
Diabetes (type 2)	21 (5.6)
Hyperlipidemia	13 (3.5)
Hypertension	74 (19.7)
Irritable bowel syndrome	41 (10.9)
Liver disease	6 (1.6)
Myocardial infarction	4 (1.1)
Peptic ulcer disease	2 (0.5)
Peripheral vascular disease	4 (1.1)
Sleep disorders (including sleep apnea)	73 (19.5)
Ulcer	10 (2.7)
**Comorbidities, ever diagnosed (n [%])**	
AIDS/HIV	2 (0.5)
Asthma	68 (18.1)
Cancer	23 (6.1)
Cerebrovascular accident or transient ischemic attack	3 (0.8)
Chronic anxiety	119 (31.7)
Chronic pulmonary disease	25 (6.7)
Congestive heart failure	6 (1.6)
Connective tissue disease	11 (2.9)
Dementia	0 (0.0)
Depression	143 (38.1)
Diabetes (type 1)	3 (0.8)
Diabetes (type 2)	23 (6.1)
Hyperlipidemia	10 (2.7)
Hypertension	79 (21.1)
Irritable bowel syndrome	47 (12.5)
Liver disease	9 (2.4)
Myocardial infarction	3 (0.8)
Peptic ulcer disease	2 (0.5)
Peripheral vascular disease	3 (0.8)
Sleep disorders (including sleep apnea)	76 (20.3)
Ulcer	13 (3.5)
**Resources used in past 12 months (n [%])**	
Primary care physician	343 (91.5)
Specialist	167 (44.5)
Urgent care facility	135 (36.0)
Emergency room visit	101 (26.9)
Hospital (admitted/hospitalized)	36 (9.6)
Other	17 (4.5)
**Mean number of resources used/visits made in past 12 months (mean [SD])**	
Primary care physician	3.7 (8.0)
Specialist	2.0 (5.6)
Urgent care facility	0.8 (2.2)
Emergency room visit	0.6 (1.7)
Hospital (admitted/hospitalized)	0.2 (1.0)
Other	0.7 (6.0)
**General health (mean [SD])**	
Height, inches	64.5 (3.0)
Weight, pounds	164.7 (47.3)
Body Mass Index, Kg/m^2^	27.8 (7.5)
Number of Days Exercised ≥ 20 Minutes in the Last Month	10.4 (9.4)

^a^ ‘Hispanic’ relates to ethnicity, and these participants could be of any race.

Acquired immune deficiency syndrome (AIDS). Human immunodeficiency virus (HIV). Standard deviation (SD). United States (US). Uncomplicated urinary tract infection (uUTI).

### Activity impairment (AIA)

**Fig 1:**
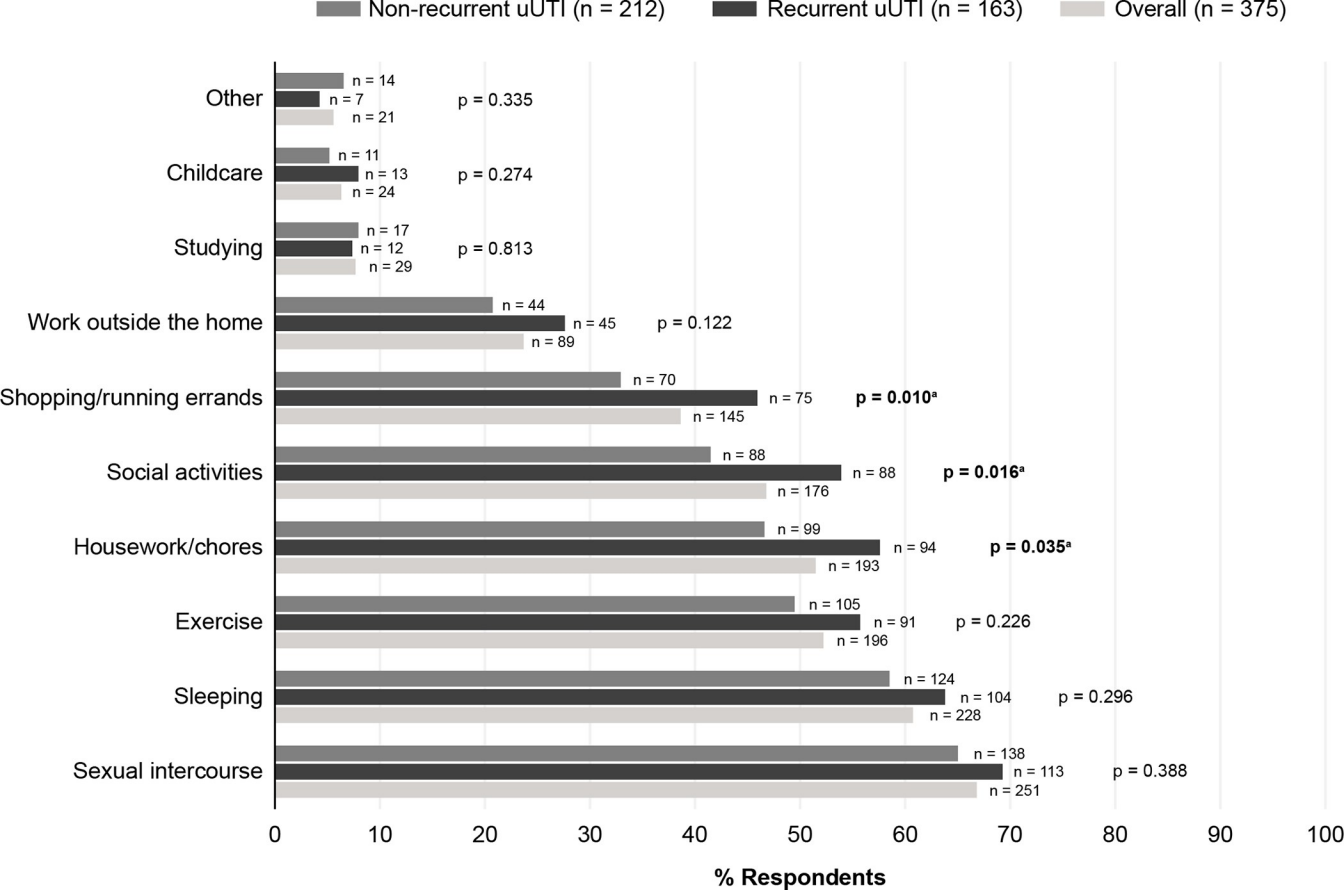
Activities impacted by uUTI. ^a^ Statistically significant difference (p < 0.05). Uncomplicated urinary tract infection (uUTI).

Commonly reported impaired activities were sexual intercourse (66.9%), sleep (60.8%), exercise (52.3%), housework/chores (51.5%), and social activities (46.9%; Fig 1). The overall mean AIA score was 11.1. Participants with recurrent uUTI reported greater impairment of activities than those with non-recurrent uUTI (Fig 1), specifically for shopping/running errands (46.0% vs. 33.0%; p = 0.010), housework/chores (57.7% vs. 46.7%; p = 0.035), and social activities (54.0% vs. 41.5%; p = 0.016).

When stratified by the number of antibiotics received, there were significant differences in the impact of uUTI on exercise (based on use of 1 vs. ≥ 3 therapies) and sleep (based on use of 2 vs. ≥ 3 therapies; Table 2).

**Table 2:** Activities and HRQoL outcomes stratified by number of oral antibiotics used to treat uUTI.

Activities impacted by uUTI, n (%)	1 AB (n = 235)	2 AB (n = 88)	≥ 3 AB (n = 52)	P-value for comparison		
				1 vs. 2 AB	1 vs. ≥ 3 AB	2 vs. ≥ 3 AB
Sexual intercourse	157 (66.8)	59 (67.0)	35 (67.3)	0.968	0.945	0.975
Sleeping	147 (62.6)	45 (51.1)	36 (69.2)	0.063	0.365	0.036^a^
Exercise	118 (50.2)	44 (50.0)	34 (65.4)	0.973	0.047^a^	0.077
Housework/chores	116 (49.4)	50 (56.8)	27 (51.9)	0.233	0.738	0.574
Social activities	106 (45.1)	45 (51.1)	25 (48.1)	0.334	0.697	0.727
Shopping/running errands	86 (36.6)	39 (44.3)	20 (38.5)	0.205	0.801	0.498
Work outside the home	51 (21.7)	26 (29.5)	12 (23.1)	0.141	0.828	0.406
Studying	15 (6.4)	7 (8.0)	7 (13.5)	0.618	0.083	0.294
Childcare	13 (5.5)	6 (6.8)	5 (9.6)	0.662	0.338	0.537
Other	13 (5.5)	5 (5.7)	3 (5.8)	1.000	1.000	1.000
PCS vs. general population^b^, n (%)						
Well below	48 (20.4)	21 (23.9)	19 (36.5)	0.548	0.030^a^	0.102
Below	43 (18.3)	19 (21.6)	5 (9.6)	–	–	–
Same or better	144 (61.3)	48 (54.5)	28 (53.8)	–	–	–
MCS vs. general population^b^, n (%)						
Well below	115 (48.9)	51 (58.0)	32 (61.5)	0.353	0.104	0.436
Below	29 (12.3)	9 (10.2)	8 (15.4)	–	–	–
Same or better	91 (38.7)	28 (31.8)	12 (23.1)	–	–	–
Mean HRQoL (SF-36v2), score (SD)						
PCS	47.3 (7.8)	45.5 (8.1)	44.6 (9.0)	0.069	0.028^a^	0.540
MCS	40.6 (12.9)	39.4 (12.6)	38.4 (11.1)	0.475	0.253	0.615
SF-6D	0.65 (0.12)	0.62 (0.12)	0.60 (0.10)	0.147	0.006^a^	0.297
AIA score, mean (SD)	11.3 (5.7)	11.2 (6.0)	10.3 (5.3)	0.908	0.234	0.355

^a^ Statistically significant difference (p < 0.05).

^b^ US average data provided by Optum Inc.

Antibiotics (AB). Activity impairment assessment (AIA). Health-related quality of life (HRQoL). Mental component score (MCS). Physical component score (PCS). Standard deviation (SD). Short form 36 version 2 (SF-36v2). Health utility index (SF-6D). Uncomplicated urinary tract infection (uUTI).

### Health-related quality of life (SF-36)

**Fig 2:**
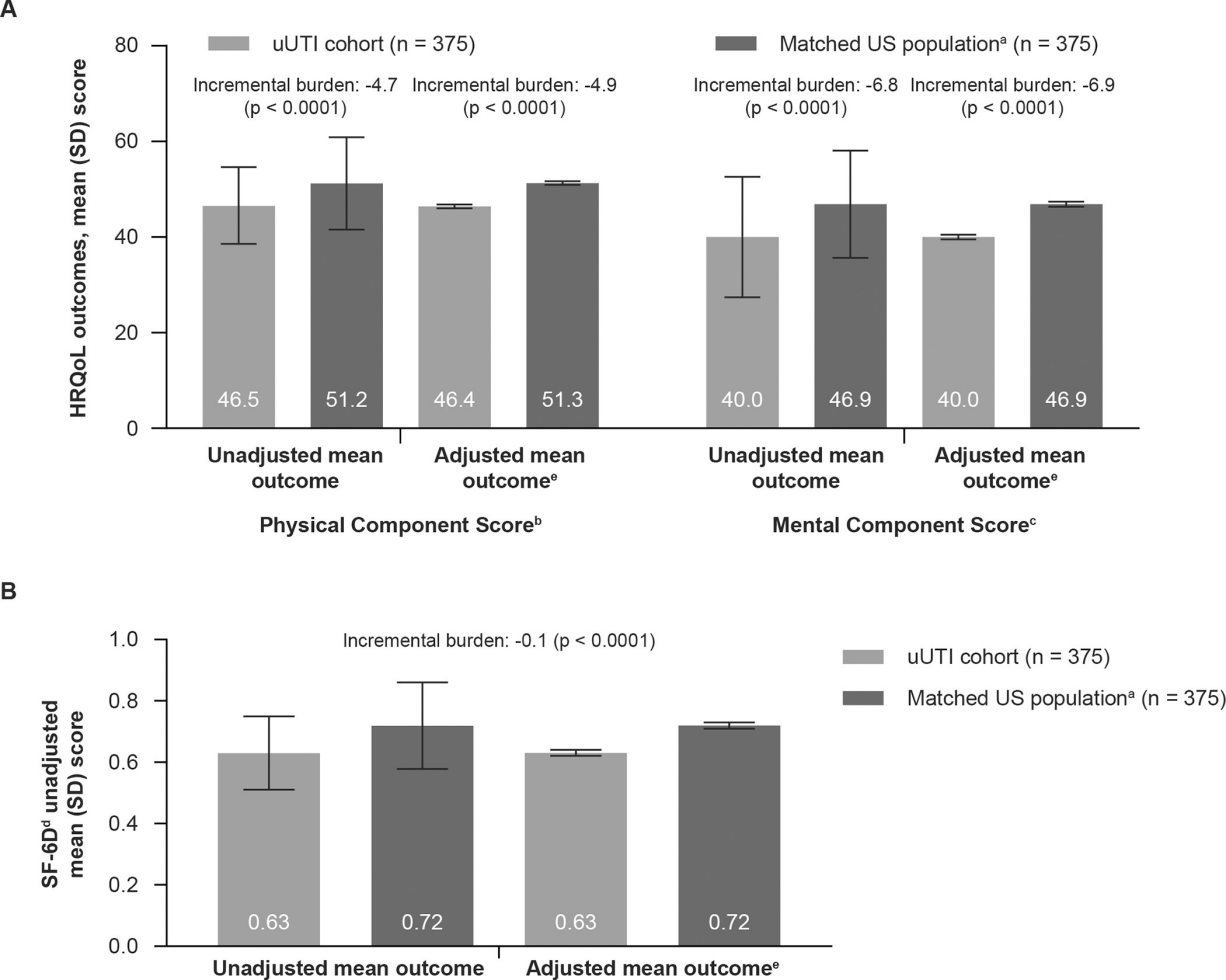
Matched analysis of SF-36v2 measured HRQoL outcomes. ^a^ Derived from the NHWS 2020. ^b^ PCS score displayed worse functioning compared to other acute infections based on US general population data provided by Optum Inc., MN, USA (e.g., acute nasopharyngitis [PCS = 48.2] and acute tracheitis [PCS = 48.4]). ^c^ MCS score at or below the same impact of both acute and chronic infections based on US general population data provided by Optum Inc., MN, USA (e.g., acute nasopharyngitis [MCS = 41.6], acute tracheitis [MCS = 41.4] and osteoarthritis [MCS = 41.7]). ^d^ A score reduction of 0.37 is above the MCID threshold for SF-6D (mean 0.041) (Walters and Brazier, 2005) [17]. ^e^ Adjusted for age, race/ethnicity, insurance, marital status, region, comorbidity count. Health-related quality of life (HRQoL). Minimal clinically important difference (MCID). Mental component score (MCS). National health and wellness survey (NHWS). Physical component score (PCS). Standard deviation (SD). Health utility index (SF-6D). Short form 36 version 2 (SF-36v2). United States (US). Uncomplicated urinary tract infection (uUTI).

Most participants (58.7%) had a PCS that was the same as or better than the general population, while for MCS, most participants (52.8%) had scores well below the general population average. Overall PCS, MCS, and SF-6D composite scores were 46.5, 40.0, and 0.63, respectively; these outcomes were significantly worse than those in the matched population (Fig 2). This was most notable for MCS where the uUTI cohort adjusted average score was 40.6 vs. the matched NHWS population adjusted score of 46.9 (Fig 2).

Participants with recurrent uUTI had worse HRQoL than those with non-recurrent uUTI. Mean PCS was 44.2 for patients with recurrent uUTI and 48.2 for those with non-recurrent uUTI (p < 0.0001). Mean SF-6D scores were 0.61 and 0.65 for recurrent and non-recurrent uUTI respectively (p = 0.004). MCS scores were similar between patients with recurrent and non-recurrent uUTI (39.1 and 40.8, respectively [p = 0.185]). Stratification by number of antibiotics used revealed statistically significant differences in the impact of uUTI on PCS and SF-6D (based on use of 1 vs. ≥ 3 therapies); Table 2).

### Work productivity and activity impairment

Mean absenteeism was 15.9, presenteeism was 50.9, overall work impairment was 56.2, and impact on daily activities was 55.0 (Table 3). Mean presenteeism was 57.6 for recurrent and 46.3 for non-recurrent uUTI (p = 0.006); for overall work impairment, mean score was 62.0 for recurrent and 52.2 for non-recurrent uUTI (p = 0.021); and for impact on daily activities, mean score was 61.3 for recurrent and 50.7 for non-recurrent uUTI (p = 0.007; Table 3).

**Table 3:** Mean WPAI outcomes overall and stratified by uUTI recurrence.

Mean (SD) WPAI, % impairment	Overall (n = 375)	Recurrent uUTI (n = 163)	Non-recurrent uUTI (n = 212)	Recurrent vs. non-recurrent, P-value
Absenteeism	15.9 (21.2)	18.6 (20.0)	14.0 (21.9)	0.144
Presenteeism	50.9 (27.8)	57.6 (25.6)	46.3 (28.4)	0.006^a^
Overall work impairment	56.2 (29.1)	62.0 (28.0)	52.2 (29.2)	0.021^a^
Impact on daily activities	55.0 (26.8)	61.3 (25.1)	50.7 (27.2)	0.007^a^

^a^ Statistically significant (p < 0.05).

Standard deviation (SD). Uncomplicated urinary tract infection (uUTI). Work productivity and activity impairment (WPAI).

Participants in the uUTI cohort reported significantly worse absenteeism (+15.3%), presenteeism (+46.5%), overall work impairment (+52.4%), and impact on daily activities (+50.7%) than the matched NHWS cohort (p < 0.0001; Table 4).

**Table 4:** Mean WPAI data for uUTI and NHWS cohorts.

WPAI Component	Mean (SD) adjusted outcomes		P-value	Incremental burden of uUTI (%)	Interpretation
	uUTI cohort	NHWS cohort			
Absenteeism	16.4 (3.4)	1.1 (0.2)	< 0.0001^a^	15.3	Approximately 6 hours of missed work in uUTI cohort
Presenteeism	53.8 (6.7)	7.4 (0.9)	< 0.0001^a^	46.5	Ability to work while working impacted by ~47% in uUTI cohort
Overall work impairment	60.6 (7.4)	8.2 (1.0)	< 0.0001^a^	52.4	Overall ability to work impacted by ~52% in uUTI cohort
Impact on daily activities	59.0 (7.1)	8.3 (1.0)	< 0.0001^a^	50.7	Overall daily activities impacted by ~51% in uUTI cohort

^a^ Statistically significant (p < 0.05).

National Health and Wellness Survey (NHWS). Standard deviation (SD). Uncomplicated urinary tract infection (uUTI). Work productivity and activity impairment (WPAI).

### Healthcare resource use

The highest reported type of HRU used for treatment of recent uUTIs were PCP visits (68.8%). There were an average of 1.46 PCP, 0.31 obstetrician/gynecologist (OB/GYN), 0.41 urgent care, and 0.08 ER visits, and 0.01 hospitalizations for participants’ most recent uUTI (Table 5).

**Table 5:** Total overall mean uUTI-related healthcare resource use, and direct and indirect costs.

	n = 375
**uUTI-related HRU, mean visits (SD) for most recent uUTI**	
Primary care physician	1.46 (5.34)
OB/GYN	0.31 (2.91)
Urgent care facility	0.41 (2.64)
ER visit	0.08 (0.34)
Hospital (admitted/hospitalized)	0.01 (0.09)
**Mean (SD) uUTI-related direct costs, $**	
Total OOP costs	90 (168)
PCP visit-related costs	491 (1828)
OB/GYN visit-related costs	105 (966)
Urgent care visit-related costs	390 (2049)
ER visit-related costs	96 (421)
Hospitalization-related costs	118 (1315)
Total direct costs	1289 (3960)
**Mean (SD) uUTI-related indirect costs, $**	
Cost of presenteeism	348 (230)
Cost of absenteeism	166 (228)
Total indirect cost	515 (311)

Emergency room (ER). Healthcare resource use (HRU). Primary care practitioner (PCP). Obstetrician/gynecologist (OB/GYN). Out of pocket (OOP). Standard deviation (SD). Uncomplicated urinary tract infection (uUTI).

### Costs

Total mean uUTI-related direct and indirect costs were $1289 and $515, respectively (Table 5). PCP and urgent care visits were the biggest contributors to direct costs ($491 and $390, respectively; Table 5). Mean total out-of-pocket costs were higher in participants with recurrent vs. non-recurrent uUTI ($112 vs. $72; p = 0.032), as were mean total indirect costs ($573 vs. $475; p *=* 0.031). Adjusted mean total direct costs were significantly higher for participants who received 2 antibiotics vs. those who received 1 ($2090 vs. $776, p < 0.0001), and for participants who received multiple antibiotics vs. those who received 1 appropriate antibiotic only ($1642 vs. $875, p = 0.002) (Table 6). Adjusted total direct costs for participants who received ≥ 3 antibiotics for their uUTI were lower than those who received 2 antibiotics ($1041 vs. $2090) and not significantly different to those who received just 1 antibiotic (p = 0.197).

**Table 6:** Estimated uUTI-related direct costs stratified by (A) number of AB and (B) appropriateness of AB therapy used to treat the last uUTI.

Stratification		Estimate (SE)	P-value	Adjusted mean costs (SE), $
Number of AB	3+ ABs, any line (n = 52)	0.29 (0.23)	0.197	1041 (215)^a^
	2 ABs, any line (n = 88)	0.99 (0.19)	< 0.0001^b^	2090 (343)^a^
	1 AB, any line (n = 235)	*Reference*	na	776 (76)^a^
Appropriateness of AB	Multiple ABs (n = 140)	0.63 (0.20)	0.002^b^	1642 (217)^c^
	1 inappropriate AB, (n = 112)	–0.26 (0.21)	0.204	673 (98)^c^
	1 appropriate AB, (n = 123)	*Reference*	na	875 (125)^c^

^a^ Costs were adjusted for age, race/ethnicity, employment and use of any second line AB.

^b^ Statistically significant (p < 0.05).

^c^ Costs were adjusted for age, race/ethnicity, employment, region, any physician-ordered urine testing. The number of antibiotics used for the most recent uUTI was based on self-report from participants; ‘1 appropriate AB’ was defined as only one first-line oral AB used (self-reported) to treat last uUTI (i.e., trimethoprim-sulfamethoxazole, nitrofurantoin, fosfomycin); ‘1 inappropriate AB’ was defined as only one second-line oral AB used (self-reported) to treat last uUTI (i.e., ciprofloxacin, ofloxacin, levofloxacin, amoxicillin-clavulanate, cefdinir, cefaclor, cefpodoxime-proxetil, cephalexin); ‘Multiple ABs’ was defined as two or more different ABs (any line) used (self-reported) for most recent uUTI.

Antibiotic(s) (AB). Not applicable (na). Standard error (SE). Uncomplicated urinary tract infection (uUTI).

### Treatment satisfaction

Treatment satisfaction results can be found in the Supplementary results. Generally, participants with non-recurrent uUTI or who received 1 antibiotic had better treatment satisfaction scores compared with participants with recurrent uUTI or who received > 1 antibiotic, respectively.

## Discussion

Among women, uUTIs are a common occurrence [1]. In our study, based on recall, 43% of women had recurrent uUTIs, which was higher than the prevalence reported in the literature, ranging from 20–40% [1,18–20]. Despite this prevalence, the burden of disease on patients is not well understood. We found that HRQoL scores, particularly MCS, were worse among participants with uUTI than a matched population based on the 2020 NHWS. Similarly, WPAI measures were significantly affected by uUTI compared with the NHWS cohort. These data demonstrate the substantial burden on patients that uUTI represents.

Furthermore, we found that survey participants with recurrent uUTI (43.5%) displayed a higher level of activity impairment (shopping, housework/chores and socialising, and WPAI impact on daily activities), worse HRQoL (PCS, SF-6D) and productivity scores (presenteeism, overall work impairment), higher mean total out-of-pocket costs, and higher mean total indirect costs compared with participants with non-recurrent uUTI.

The burden of recurrent UTI from the patient perspective was previously described in a qualitative study of comments on an online forum which demonstrated the substantial and varied impact that repeated infections have on individuals’ quality of life [21]. As with our study, these authors found sexual intercourse to be a commonly affected activity [21]. Furthermore, an online survey of women with recurrent UTI in 5 European countries found that 23–55% of participants (depending on country and whether the participants had a current acute UTI or had a UTI in the past 4 weeks) had physical health scores below a US general population comparator, and 55–81% had mental health scores below the comparator [22]. This is consistent with our study where a greater proportion of the cohort had MCS scores below the general population than PCS scores. However, our study has the strength of providing a comparison between recurrent and non-recurrent uUTI, allowing us to demonstrate the additional HRQoL burden that patients with recurrent uUTI have above and beyond that conferred by the infection alone. Additionally, many of the participants in the previous studies would have been ineligible for this study due to signs of complicated UTI. By including just participants with uUTI, we were able to show that even with infections that might conventionally be considered mild, a significant burden is experienced by patients that affects HRQoL.

When stratified by number of oral antibiotics used, participants who received ≥ 3 antibiotics for their most recent uUTI reported worse HRQoL (PCS, SF-6D) than those who received only 1 antibiotic. This could be due to these participants having more severe disease or receiving inappropriate treatment resulting in more prescriptions, thereby resulting in increased patient burden. However, our methodology excluded participants with more severe UTI and asymptomatic bacteriuria, yielding a population with symptomatic uUTI. While we did not exclude participants with concurrent infections, the survey questions were specifically related to their uUTI.

Similarly, when stratified by clinically appropriate oral antibiotic use, participants who received multiple antibiotics had worse HRQoL (PCS, SF-6D) and a higher level of activity impairment (WPAI impact on daily activities) than those who received 1 appropriate antibiotic, and a greater mean total direct cost than those who received 1 inappropriate antibiotic.

In terms of treatment satisfaction, we found that participants with recurrent uUTIs who received more than 1 antibiotic were less satisfied with treatment than those with non-recurrent uUTIs treated successfully with 1 antibiotic. These data paralleled other endpoints, i.e., recurrent uUTI and more antibiotics were associated with worse outcomes. High levels of satisfaction associated with single antibiotic therapy have been previously reported [23].

The increased direct uUTI-related costs that we observed with the use of multiple vs. single oral antibiotics could be due to inefficient prescribing. Inappropriate prescription of antibiotics based on drug class and duration of therapy has previously been shown to be prevalent in the treatment of uUTI [24]. Our results, however, suggest that ineffective therapies are being prescribed as patients require multiple antibiotics to resolve their uUTIs. Thus, identification of the most appropriate antibiotic therapy may help to optimize direct uUTI-related costs associated with antibiotic treatment. Use of multiple antibiotics can also increase the risk of antimicrobial resistance developing, which is an increasing problem globally [6], and not consistent with antibiotic stewardship practices [25,26]. *E*. *coli*, the predominant uropathogen responsible for uUTI [1], is a common causative pathogen for other diseases and a World Health Organization priority pathogen, identified at critical risk of antimicrobial resistance [6].

The most common healthcare resource used by participants to treat their recent uUTI were primary care physician visits followed by urgent care visits and OB/GYN visits. The majority of total direct costs were due to PCP visits and urgent care visits. Compared with the general population, uUTI had significant impacts on absenteeism and presenteeism, resulting in indirect costs that most directly impact employers, specifically those that provide health insurance to employees (70% of matched sample). Insurers that provide coverage for employers and employers who contract insurers for employer-sponsored healthcare plans should account for indirect costs related to uUTI episodes, in addition to the direct treatment costs.

These findings are consistent with earlier work in women with UTI. For example, a prospective survey of women with UTI in England found similar rates of HRU, with 65% of patients who contacted a healthcare professional going to primary care, 4% attending an emergency department and 14% contacting an out-of-hours service [3]. In accordance with our study, an observational study conducted across multiple countries found that anxiety and depression were the most commonly reported comorbidities among women with uUTIs at baseline [27]. Furthermore, the HRQoL scores (PCS and MCS) reported in our study were similar to those reported in a French study of patients with cystitis or other female genital diseases; with a mean PCS of 45.6 and MCS of 41.5 compared with 46.5 and 40.0 in the current study, respectively [28].

A strength of this study is that results are generalizable to adult women in the US diagnosed with uUTI, and as discussed above are similar to results from other regions. There are, however, a number of study limitations. The main limitation relates to the nature of the data. Self-report for uUTI carries the innate possibility of recall bias which could affect the results, particularly those regarding out-of-pocket costs and recent treatments. Recall bias could also have allowed for the inclusion of a higher proportion of women with recurrent uUTIs. Additionally, we were not able to capture the rationale behind the decisions to prescribe certain antibiotics, and thus second-line therapies that were selected for appropriate reasons would nevertheless have been classed as inappropriate in our study. We also adjusted the recall period for key instrument measures used in the study. Validated versions of SF-36, WPAI and AIA are based on recall periods of the previous 4 weeks, 7 days and 24 hours, respectively; we used up to 60 days and adjustment to a longer look-back period may have affected the accuracy and validity of these instrument outcomes. We altered the lookback period both to standardize the instruments to a single period and to allow us to capture a large enough patient population, where the shorter time periods would have restricted eligible responders to those who had a uUTI in the previous 24 hours. Additionally, the surveys were only conducted in English which limited the diversity of the population available for the study. Total, direct uUTI-related HRU costs were based on imputation using mean estimates per event type from the 2018 Medical Expenditure Panel Survey. This cost imputation, along with the previously mentioned limitation associated with self-reported HRU, may be factors in the finding from the regression analysis that participants who self-reported using 2 antibiotics for their recent uUTI-related treatment had uUTI-related direct costs roughly double that of participants reporting use of ≥ 3 antibiotics for their recent uUTI. As such, these do not represent actual adjudicated costs, and this should be noted when interpreting the results. Additionally, participants were not excluded for having concurrent other infections and may have erroneously self-reported use of antibiotics for uUTI which were actually used for another condition. A further limitation of this survey is that it is only representative of patients willing to participate, perhaps women with recurrent uUTI, and will therefore not capture the disease burden, perceptions, and unmet needs of those not willing to participate.

## Conclusions

This study demonstrates that uUTIs are significantly associated with worse patient-reported outcomes such as daily activities, work productivity and mental HRQoL, and that suboptimal treatment (i.e., use of multiple antibiotics) may play a role. Inadequate treatment response, evident by use of multiple antibiotics to treat a uUTI, was associated with an increase in uUTI-related costs, including productivity loss. While uUTIs are common, their impact on patients should not be underestimated; appropriate treatment is crucial in preventing adverse impacts on quality-of-life and HRU.

## Supporting information

S1 Dataset (XLSX)

S1 File (DOCX)

## References

[1] Medina M, Castillo-Pino E. An introduction to the epidemiology and burden of urinary tract infections. Ther Adv Urol. 2019;11:1756287219832172. doi: 10.1177/1756287219832172 31105774 PMC6502976

[2] Mehnert-Kay SA. Diagnosis and management of uncomplicated urinary tract infections. Am Fam Physician. 2005;72(3):451–6. 16100859

[3] Butler CC, Hawking MK, Quigley A, McNulty CA. Incidence, severity, help seeking, and management of uncomplicated urinary tract infection: a population-based survey. Br J Gen Pract. 2015;65(639):e702–7. doi: 10.3399/bjgp15X686965 26412847 PMC4582883

[4] Schappert SM, Rechtsteiner EA. Ambulatory medical care utilization estimates for 2007. National Center for Health Statistics. Vital Health Stat 13. 2011(169):1–38. 21614897

[5] Gupta K, Hooton TM, Naber KG, Wult B, Colgan R, Miller LG, et al. International clinical practice guidelines for the treatment of acute uncomplicated cystitis and pyelonephritis in women: a 2010 update by the Infectious Diseases Society of America and the European Society for Microbiology and Infectious Diseases. Clin Infect Dis. 2011;52(5):e103–20. doi: 10.1093/cid/ciq257 21292654

[6] World Health Organization. WHO publishes list of bacteria for which new antibiotics are urgently needed. 2017. Available from: https://www.who.int/news/item/27-02-2017-who-publishes-list-of-bacteria-for-which-new-antibiotics-are-urgently-needed (Accessed December 2021).

[7] Öztürk R, Murt A. Epidemiology of urological infections: a global burden. World J Urol. 2020;38(11):2669–79. doi: 10.1007/s00345-019-03071-4 31925549

[8] Ellis AK, Verma S. Quality of life in women with urinary tract infections: is benign disease a misnomer? J Am Board Fam Pract. 2000;13(6):392–7. doi: 10.3122/15572625-13-6-392 11117334

[9] Anger J, Lee U, Ackerman AL, Chou R, Chughtai B, Clemens JQ, et al. Recurrent uncomplicated urinary tract infections in women: AUA/CUA/SUFU guideline. J Urol. 2019;202(2):282–9. doi: 10.1097/JU.0000000000000296 31042112

[10] Wild DJ, Clayson DJ, Keating K, Gondek K. Validation of a patient-administered questionnaire to measure the activity impairment experienced by women with uncomplicated urinary tract infection: the Activity Impairment Assessment (AIA). Health Qual Life Outcomes. 2005;3:42. doi: 10.1186/1477-7525-3-42 16022727 PMC1180845

[11] Maruish ME, Kosinski M, Bjorner JB, Gandek B, Turner-Bowker DM, Ware JE. User’s manual for the SF36v2 Health Survey, Lincoln, Quality Metric. 2011.

[12] Medical Expenditure Panel Survey (MEPS). Mean expenditure per event by event type and age groups, United States, 2018. Agency for Healthcare Research and Quality, Rockville, MD. Accessed from: https://datatools.ahrq.gov/meps-hc.

[13] Reilly MC, Zbrozek AS, Dukes EM. 1993. The validity and reproducibility of a work productivity and activity impairment instrument. Pharmacoeconomics. 1993;4(5):353–65. doi: 10.2165/00019053-199304050-00006 10146874

[14] Bureau of Labor Statistics. Medical care in U.S. city average, all urban consumers, not seasonally adjusted; 2018–2020. Series ID CUUR0000SAM. Accessed from: https://data.bls.gov/pdq/SurveyOutputServlet.

[15] Hanmer J, Kaplan RM. Update to the report of nationally representative values for the noninstitutionalized US adult population for five health-related quality-of-life scores. Value Health. 2016;19(8):1059–62. doi: 10.1016/j.jval.2016.05.019 27987633 PMC5408863

[16] Cerner Envisia. 2020. National health and wellness survey [Online]. New York. Available from: https://www.cernerenviza.com/real-world-data/national-health-and-wellness-survey-nhws [Accessed 14 December 2021].

[17] Walters SJ, Brazier JE. Comparison of the minimally important difference for two health state utility measures: EQ-5D and SF-6D. Qual Life Res. 2005 Aug;14(6):1523–32. doi: 10.1007/s11136-004-7713-0 16110932

[18] Foxman B. Recurring urinary tract infection: incidence and risk factors. Am J Public Health. 1990;80(3):331–3. doi: 10.2105/ajph.80.3.331 2305919 PMC1404686

[19] Ikäheimo R, Siitonen A, Heiskanen T, Kärkkäinen U, Kuosmanen P, Lipponen P, et al. Recurrence of urinary tract infection in a primary care setting: analysis of a 1-year follow-up of 179 women. Clin Infect Dis. 1996;22(1):91–9. doi: 10.1093/clinids/22.1.91 8824972

[20] Gupta K, Trautner BW. Diagnosis and management of recurrent urinary tract infections in non-pregnant women. BMJ. 2013;346:f3140. doi: 10.1136/bmj.f3140 23719637 PMC4688544

[21] Flower A, Bishop FL, Lewith G. How women manage recurrent urinary tract infections: an analysis of postings on a popular web forum. BMC Fam Pract. 2014;15:162. doi: 10.1186/1471-2296-15-162 25260870 PMC4262982

[22] Wagenlehner F, Wullt B, Ballarini S, Zingg D, Naber KG. Social and economic burden of recurrent urinary tract infections and quality of life: a patient web-based study (GESPRIT). Expert Rev Pharmacoecon Outcomes Res. 2018;18:107–17. doi: 10.1080/14737167.2017.1359543 28737469

[23] Nickel JC, Lee JC, Grantmyre JE, Polygenis D. Natural history of urinary tract infection in a primary care environment in Canada. Can J Urol. 2005;12(4):2728–37. 16197596

[24] Durkin MJ, Keller M, Butler AM, Kwon JH, Dubberke ER, Miller AC, et al. An assessment of inappropriate antibiotic use and guideline adherence for uncomplicated urinary tract infections. Open Forum Infect Dis. 2018;5(9):ofy198. doi: 10.1093/ofid/ofy198 30191156 PMC6121225

[25] The Joint Commission. New antimicrobial stewardship standard. Available from: https://www.jointcommission.org/assets/1/6/New_Antimicrobial_Stewardship_Standard.pdf. Accessed December 2021.

[26] Centers for Disease Control and Prevention. The core elements of outpatient antibiotic stewardship. Available from: https://www.cdc.gov/antibiotic-use/core-elements/outpatient.html. Accessed 9 November 2021.

[27] Renard J, Ballarini S, Mascarenhas T, Zahran M, Quimper E, Choucair J, et al. Recurrent lower urinary tract infections have a detrimental effect on patient quality of life: a prospective, observational study. Infect Dis Ther. 2014;4(1):125–35. doi: 10.1007/s40121-014-0054-6 25519161 PMC4363217

[28] Grimaldi-Bensouda L, Begaud B, Lert F, Rouillon F, Massol J, Guillemot D, et al. Benchmarking the burden of 100 diseases: results of a nationwide representative survey within general practices. BMJ Open. 2011;1(2):e000215. doi: 10.1136/bmjopen-2011-000215 22102638 PMC3221295

