# Supplementary material for: Activity impairment, health-related quality of life, productivity, and self-reported resource use and associated costs of uncomplicated urinary tract infection among women in the United States
Source: PLoS One. 2023 Feb 1;18(2):e0277728. doi: 10.1371/journal.pone.0277728 (PMC9891499; doi:10.1371/journal.pone.0277728)
Supplement: S1 File — (DOCX) [file pone.0277728.s002.docx]

# Supporting information

## Supplementary methods

**S1 Table. Demographic distribution of panel populations compared to 2019 US census.**

|  | **2019 US census (%)** | **2019 Kantar profiles (%)** | **Dynata 2019 (%)** | **EMI 2019  (%)** | **Lucid/ federated (%)** |
| --- | --- | --- | --- | --- | --- |
| **Age, years** |  |  |  |  |  |
| 18–24 | 9 | 23 | 16 | 22 | 11 |
| 25–34 | 13 | 26 | 24 | 27 | 26 |
| 35–44 | 13 | 18 | 19 | 19 | 21 |
| 45–64 | 25 | 23 | 28 | 16 | 31 |
| 65+ | 16 | 7 | 10 | 15 | 10 |
| **Sex** |  |  |  |  |  |
| Female | 51 | 66 | 65 | 60 | 59 |
| **Region** |  |  |  |  |  |
| Northeast | 17 | 14 | 19 | Not available | Not available |
| Midwest | 21 | 21 | 20 | Not available | Not available |
| South | 38 | 44 | 40 | Not available | Not available |
| West | 24 | 21 | 21 | Not available | Not available |

United States (US).

*Treatment satisfaction*

Treatment satisfaction was assessed using the Treatment Satisfaction Questionnaire for Medication (TSQM-9). Participants were instructed to complete the TSQM based on the most recent oral antibiotic medication taken for the treatment of their uncomplicated urinary tract infection (uUTI).

The TSQM comprises three scales measuring satisfaction with medication effectiveness and convenience, and global satisfaction [1]. The Effectiveness and Convenience scales (three items each, using 7-point Likert scale responses) include items such as, “How satisfied or dissatisfied are you with the ability of the medication to prevent or treat your condition?” (Effectiveness) and, “How easy or difficult is it to use the medication in its current form?” (Convenience), with higher scores indicating greater satisfaction. The Global Satisfaction scale (three items, summing across 5-point Likert scale responses) of the TSQM is used to assess the overall level of satisfaction or dissatisfaction; higher scores indicate greater satisfaction.

## Supplementary results

**S2 Table. Demographic characteristics stratified by number of oral antibiotics used to treat last uUTI.**

|  | **1 AB (n = 235)** | **2 ABs (n = 88)** | **≥ 3 ABs (n = 52)** | **P-value for comparison** | | |
| --- | --- | --- | --- | --- | --- | --- |
|  |  |  |  | **1 vs. 2 AB** | **1 vs. ≥ 3 AB** | **2 vs. ≥ 3 AB** |
| **Age, years (n [%])** |  |  |  |  |  |  |
| 18–29 | 43 (18.3) | 13 (14.8) | 10 (19.2) | 0.728 | 0.978 | 0.883 |
| 30–39 | 55 (23.4) | 27 (30.7) | 13 (25.0) |  |  |  |
| 40–49 | 49 (20.9) | 16 (18.2) | 11 (21.2) |  |  |  |
| 50–64 | 55 (23.4) | 20 (22.7) | 10 (19.2) |  |  |  |
| 65+ | 33 (14.0) | 12 (13.6) | 8 (15.4) |  |  |  |
| **Insurance type (n [%])** |  |  |  |  |  |  |
| Employer Provided | 104 (44.3) | 31 (35.2) | 19 (36.5) | 0.093 | 0.818 | 0.952 |
| State Health Exchange ('Obamacare') | 19 (8.1) | 4 (4.5) | 3 (5.8) |  |  |  |
| Dual | 4 (1.7) | 6 (6.8) | 2 (3.8) |  |  |  |
| Medicare | 50 (21.3) | 18 (20.5) | 13 (25.0) |  |  |  |
| Medicaid | 33 (14.0) | 19 (21.6) | 10 (19.2) |  |  |  |
| No Insurance | 14 (6.0) | 4 (4.5) | 3 (5.8) |  |  |  |
| Other | 11 (4.7) | 6 (6.8) | 2 (3.8) |  |  |  |
| **Marital status (n [%])** |  |  |  |  |  |  |
| Married | 112 (47.7) | 33 (37.5) | 20 (38.5) | 0.165 | 0.073 | 0.456 |
| Single, never married | 42 (17.9) | 20 (22.7) | 11 (21.0) |  |  |  |
| Divorced | 38 (16.2) | 18 (20.5) | 7 (13.5) |  |  |  |
| Separated | 2 (0.9) | 3 (3.4) | 4 (7.7) |  |  |  |
| Widowed | 10 (4.3) | 7 (8.0) | 2 (3.8) |  |  |  |
| Living with partner | 29 (12.3) | 7 (8.0) | 8 (15.4) |  |  |  |
| Other/Not reported | 2 (0.9) | 0 | 0 |  |  |  |
| **Race/Ethnicity (n [%])** |  |  |  |  |  |  |
| African American/Black | 8 (3.4) | 4 (4.5) | 1 (1.9) | 0.985 | 0.766 | 0.725 |
| Asian or Pacific Islander | 7 (3.0) | 2 (2.3) | 2 (3.8) |  |  |  |
| Hispanic | 14 (6.0) | 5 (5.7) | 3 (5.8) |  |  |  |
| White | 200 (85.1) | 75 (85.2) | 43 (82.7) |  |  |  |
| Multiple/Other | 6 (2.6) | 2 (2.3) | 3 (5.8) |  |  |  |
| **Employment type (n [%])** |  |  |  |  |  |  |
| Full-time | 91 (38.7) | 24 (27.3) | 13 (25.0) | 0.515 | 0.530 | 0.815 |
| Part-time | 22 (9.4) | 9 (10.2) | 7 (13.5) |  |  |  |
| Self-employed | 9 (3.8) | 2 (2.3) | 1 (1.9) |  |  |  |
| Homemaker | 27 (11.5) | 13 (14.8) | 11 (21.2) |  |  |  |
| Retired | 35 (14.9) | 14 (15.9) | 9 (17.3) |  |  |  |
| Unemployed | 30 (12.8) | 14 (15.9) | 6 (11.5) |  |  |  |
| Disabled | 14 (6.0) | 10 (11.4) | 3 (5.8) |  |  |  |
| Student | 5 (2.1) | 2 (2.3) | 1 (1.9) |  |  |  |
| [Decline to answer] | 2 (0.9) | 0 | 1 (1.9) |  |  |  |
| **Education (n [%])** |  |  |  |  |  |  |
| Less than high school | 2 (0.9) | 1 (1.1) | 0 | 0.468 | 0.922 | 0.634 |
| Completed some high school | 8 (3.4) | 4 (4.5) | 1 (1.9) |  |  |  |
| High school graduate or equivalent (e.g., GED) | 34 (14.5) | 12 (13.6) | 9 (17.3) |  |  |  |
| Completed some college, but no degree | 59 (25.1) | 33 (37.5) | 14 (26.9) |  |  |  |
| Associate’s degree | 37 (15.7) | 13 (14.8) | 9 (17.3) |  |  |  |
| College graduate (e.g., B.A., A.B., B.S.) | 65 (27.7) | 15 (17.0) | 11 (21.2) |  |  |  |
| Completed some graduate school, but no degree | 8 (3.4) | 4 (4.5) | 1 (1.9) |  |  |  |
| Completed graduate school (e.g., M.S., M.D., Ph.D.) | 21 (8.9) | 6 (6.8) | 7 (13.5) |  |  |  |
| [Decline to answer] | 1 (0.4) | 0 | 0 |  |  |  |
| **Residence (n [%])** |  |  |  |  |  |  |
| Urban/City | 51 (21.7) | 17 (19.3) | 20 (38.5) | 0.305 | 0.035^a^ | 0.018^a^ |
| Suburban | 129 (54.9) | 43 (48.9) | 24 (46.2) |  |  |  |
| Rural | 55 (23.4) | 28 (31.8) | 8 (15.4) |  |  |  |
| **US Region (n [%])** |  |  |  |  |  |  |
| Northeast | 56 (23.8) | 12 (13.6) | 7 (13.5) | 0.044^a^ | 0.100 | 0.992 |
| South | 53 (22.6) | 14 (15.9) | 8 (15.4) |  |  |  |
| Midwest | 90 (38.3) | 47 (53.4) | 29 (55.8) |  |  |  |
| West | 36 (15.3) | 15 (17.0) | 8 (15.4) |  |  |  |
| **Household income (n [%])** |  |  |  |  |  |  |
| Less than $15,000 | 19 (8.1) | 13 (14.8) | 5 (9.6) | 0.143 | 0.118 | 0.801 |
| $15,000 to $24,999 | 20 (8.5) | 5 (5.7) | 6 (11.5) |  |  |  |
| $25,000 to $34,999 | 17 (7.2) | 10 (11.4) | 6 (11.5) |  |  |  |
| $35,000 to $49,999 | 31 (13.2) | 10 (11.4) | 6 (11.5) |  |  |  |
| $50,000 to $74,999 | 42 (17.9) | 23 (26.1) | 15 (28.8) |  |  |  |
| $75,000 to $99,999 | 45 (19.1) | 12 (13.6) | 3 (5.8) |  |  |  |
| $100,000 to $124,999 | 20 (8.5) | 8 (9.1) | 7 (13.5) |  |  |  |
| $125,000 to $149,999 | 13 (5.5) | 4 (4.5) | 3 (5.8) |  |  |  |
| $150,000+ | 23 (9.8) | 2 (2.3) | 1 (1.9) |  |  |  |
| [Decline to answer] | 5 (2.1) | 1 (1.1) | 0 |  |  |  |
| **Smoking Status** |  |  |  |  |  |  |
| Never | 114 (48.5) | 36 (40.9) | 22 (42.3) | 0.561 | 0.302 | 0.524 |
| Used To | 56 (23.8) | 23 (26.1) | 9 (17.3) |  |  |  |
| Once a month or less | 4 (1.7) | 4 (4.5) | 3 (5.8) |  |  |  |
| 2-3 times per month | 3 (1.3) | 1 (1.1) | 2 (3.8) |  |  |  |
| Once a week | 2 (0.9) | 1 (1.1) | 0 |  |  |  |
| 2-3 times per week | 2 (0.9) | 3 (3.4) | 0 |  |  |  |
| 4-6 times per week | 6 (2.6) | 2 (2.3) | 3 (5.8) |  |  |  |
| Daily | 48 (20.4) | 18 (20.5) | 13 (25.0) |  |  |  |
| **Alcohol Use** |  |  |  |  |  |  |
| Never | 24 (10.2) | 13 (14.8) | 3 (5.8) | 0.490 | 0.105 | 0.178 |
| Used To | 41 (17.4) | 20 (22.7) | 8 (15.4) |  |  |  |
| Once a month or less | 60 (25.5) | 20 (22.7) | 8 (15.4) |  |  |  |
| 2-3 times per month | 32 (13.6) | 10 (11.4) | 11 (21.2) |  |  |  |
| Once a week | 32 (13.6) | 11 (12.5) | 11 (21.2) |  |  |  |
| 2-3 times per week | 32 (13.6) | 6 (6.8) | 4 (7.7) |  |  |  |
| 4-6 times per week | 7 (3.0) | 5 (5.7) | 2 (3.8) |  |  |  |
| Daily | 7 (3.0) | 3 (3.4) | 5 (9.6) |  |  |  |
| **Comorbidities (current)** |  |  |  |  |  |  |
| AIDS/HIV | 1 (0.4) | 1 (1.1) | 0 | 0.471 | 1.000 | 1.000 |
| Asthma | 43 (18.3) | 16 (18.2) | 10 (19.2) | 0.981 | 0.875 | 0.877 |
| Cancer | 13 (5.5) | 3 (3.4) | 5 (9.6) | 0.571 | 0.338 | 0.147 |
| Cerebrovascular accident or transient ischemic attack | 2 (0.9) | 2 (2.3) | 1 (1.9) | 0.300 | 0.452 | 1.000 |
| Chronic anxiety | 69 (29.4) | 26 (29.5) | 26 (50.0) | 0.974 | 0.004^a^ | 0.016^a^ |
| Chronic pulmonary disease (e.g., COPD, emphysema, chronic bronchitis) | 23 (9.8) | 5 (5.7) | 2 (3.8) | 0.276 | 0.274 | 1.000 |
| Congestive heart failure | 4 (1.7) | 0 | 0 | 0.578 | 1.000 | – |
| Connective tissue disease (e.g., rheumatoid arthritis) | 4 (1.7) | 1 (1.1) | 3 (5.8) | 1.000 | 0.115 | 0.145 |
| Dementia | 1 (0.4) | 0 | 0 | 1.000 | 1.000 | – |
| Depression | 80 (34.0) | 35 (39.8) | 22 (42.3) | 0.338 | 0.260 | 0.768 |
| Diabetes (type 1) | 0 | 1 (1.1) | 1 (1.9) | 0.272 | 0.181 | 1.000 |
| Diabetes (type 2) | 13 (5.5) | 4 (4.5) | 4 (7.7) | 1.000 | 0.522 | 0.469 |
| Hyperlipidemia | 9 (3.8) | 3 (3.4) | 1 (1.9) | 1.000 | 0.696 | 1.000 |
| Hypertension | 49 (20.9) | 16 (18.2) | 9 (17.3) | 0.594 | 0.565 | 0.896 |
| Irritable bowel syndrome | 19 (8.1) | 16 (18.2) | 6 (11.5) | 0.009^a^ | 0.419 | 0.297 |
| Liver disease | 3 (1.3) | 3 (3.4) | 0 | 0.351 | 1.000 | 0.295 |
| Myocardial infarction | 3 (1.3) | 0 | 1 (1.9) | 0.565 | 0.553 | 0.371 |
| Peptic ulcer disease | 0 | 2 (2.3) | 0 | 0.074 | – | 0.530 |
| Peripheral vascular disease | 2 (0.9) | 1 (1.1) | 1 (1.9) | 1.000 | 0.452 | 1.000 |
| Sleep disorders (including sleep apnea) | 42 (17.9) | 18 (20.5) | 13 (25.0) | 0.595 | 0.237 | 0.531 |
| Ulcer | 5 (2.1) | 3 (3.4) | 2 (3.8) | 0.454 | 0.614 | 1.000 |
| **Comorbidities (ever diagnosed)** |  |  |  |  |  |  |
| AIDS/HIV | 1 (0.4) | 1 (1.1) | 0 | 0.471 | 1.000 | 1.000 |
| Asthma | 42 (17.9) | 16 (18.2) | 10 (19.2) | 0.949 | 0.818 | 0.877 |
| Cancer | 12 (5.1) | 4 (4.5) | 7 (13.5) | 1.000 | 0.057 | 0.100 |
| Cerebrovascular accident or transient ischemic attack | 1 (0.4) | 2 (2.3) | 0 | 0.181 | 1.000 | 0.530 |
| Chronic anxiety | 68 (28.9) | 27 (30.7) | 24 (46.2) | 0.759 | 0.016^a^ | 0.066 |
| Chronic pulmonary disease (e.g., COPD, emphysema, chronic bronchitis) | 18 (7.7) | 5 (5.7) | 2 (3.8) | 0.634 | 0.546 | 1.000 |
| Congestive heart failure | 5 (2.1) | 1 (1.1) | 0 | 1.000 | 0.589 | 1.000 |
| Connective tissue disease (e.g., rheumatoid arthritis) | 7 (3.0) | 1 (1.1) | 3 (5.8) | 0.688 | 0.395 | 0.145 |
| Dementia | 0 | 0 | 0 | – | – | – |
| Depression | 85 (36.2) | 34 (38.6) | 24 (46.2) | 0.699 | 0.180 | 0.383 |
| Diabetes (type 1) | 1 (0.4) | 1 (1.1) | 1 (1.9) | 0.471 | 0.330 | 1.000 |
| Diabetes (type 2) | 13 (5.5) | 5 (5.7) | 5 (9.6) | 1.000 | 0.338 | 0.500 |
| Hyperlipidemia | 7 (3.0) | 3 (3.4) | 0 | 1.000 | 0.358 | 0.295 |
| Hypertension | 51 (21.7) | 18 (20.5) | 10 (19.2) | 0.808 | 0.693 | 0.861 |
| Irritable bowel syndrome | 25 (10.6) | 15 (17.0) | 7 (13.5) | 0.120 | 0.558 | 0.573 |
| Liver disease | 5 (2.1) | 3 (3.4) | 1 (1.9) | 0.454 | 1.000 | 1.000 |
| Myocardial infarction | 2 (0.9) | 1 (1.1) | 0 | 1.000 | 1.000 | 1.000 |
| Peptic ulcer disease | 1 (0.4) | 1 (1.1) | 0 | 0.471 | 1.000 | 1.000 |
| Peripheral vascular disease | 2 (0.9) | 1 (1.1) | 0 | 1.000 | 1.000 | 1.000 |
| Sleep disorders (including sleep apnea) | 42 (17.9) | 21 (23.9) | 13 (25.0) | 0.226 | 0.237 | 0.880 |
| Ulcer | 9 (3.8) | 1 (1.1) | 3 (5.8) | 0.297 | 0.461 | 0.145 |
| **Resources Used in Past 12 Months** |  |  |  |  |  |  |
| Primary care physician | 215 (91.5) | 81 (92.0) | 47 (90.4) | 0.872 | 0.798 | 0.761 |
| Specialist | 100 (42.6) | 42 (47.7) | 25 (48.1) | 0.404 | 0.467 | 0.968 |
| Urgent care facility | 81 (34.5) | 34 (38.6) | 20 (38.5) | 0.486 | 0.585 | 0.984 |
| Emergency room visit | 54 (23.0) | 33 (37.5) | 14 (26.9) | 0.009^a^ | 0.545 | 0.200 |
| Hospital (admitted/hospitalized) | 19 (8.1) | 12 (13.6) | 5 (9.6) | 0.132 | 0.718 | 0.597 |
| Other | 11 (4.7) | 3 (3.4) | 3 (5.8) | 0.766 | 0.724 | 0.670 |

^a^ Statistically significant difference (p < 0.05).
Acquired Immunodeficiency Syndrome (AIDS). Antibiotic (AB). Chronic Obstructive Pulmonary Disease (COPD). Human Immunodeficiency Virus (HIV). General Educational Development (GED). United States (US). Uncomplicated urinary tract infection (uUTI).

**S3 Table. Demographic characteristics stratified by clinically appropriate oral antibiotic treatment for last uUTI.**

|  | **1 appropriate AB (n = 123)** | **1 inappropriate AB (n = 112)** | **Multiple ABs  (n = 140)** | **P-value for comparison** | | |
| --- | --- | --- | --- | --- | --- | --- |
|  |  |  |  | **Appropriate vs. inappropriate** | **Appropriate vs. multiple** | **Inappropriate vs. multiple** |
| **Age, years (n [%])** |  |  |  |  |  |  |
| 18-29 | 18 (14.6) | 25 (22.3) | 23 (16.4) | 0.263 | 0.895 | 0.443 |
| 30-39 | 33 (26.8) | 22 (19.6) | 40 (28.6) |  |  |  |
| 40-49 | 23 (18.7) | 26 (23.2) | 27 (19.3) |  |  |  |
| 50-64 | 33 (26.8) | 22 (19.6) | 30 (21.4) |  |  |  |
| 65+ | 16 (13.0) | 17 (15.2) | 20 (14.3) |  |  |  |
| **Insurance type (n [%])** |  |  |  |  |  |  |
| Employer Provided | 60 (48.8) | 44 (39.3) | 50 (35.7) | 0.049^a^ | 0.309 | 0.048^a^ |
| State Health Exchange ('Obamacare') | 5 (4.1) | 14 (12.5) | 7 (5.0) |  |  |  |
| Dual | 2 (1.6) | 2 (1.8) | 8 (5.7) |  |  |  |
| Medicare | 24 (19.5) | 26 (23.2) | 41 (29.3) |  |  |  |
| Medicaid | 20 (16.3) | 13 (11.6) | 29 (20.7) |  |  |  |
| No Insurance | 4 (3.3) | 10 (8.9) | 7 (5.0) |  |  |  |
| Other | 8 (6.5) | 3 (2.7) | 8 (5.7) |  |  |  |
| **Marital status (n [%])** |  |  |  |  |  |  |
| Married | 61 (49.6) | 51 (45.5) | 53 (37.9) | 0.867 | 0.111 | 0.414 |
| Single, never married | 20 (16.3) | 22 (19.6) | 31 (22.1) |  |  |  |
| Divorced | 19 (15.4) | 19 (17.0) | 25 (17.9) |  |  |  |
| Separated | 1 (0.8) | 1 (0.9) | 7 (5.0) |  |  |  |
| Widowed | 5 (4.1) | 5 (4.5) | 9 (6.4) |  |  |  |
| Living with partner | 15 (12.2) | 14 (12.5) | 15 (10.7) |  |  |  |
| Other/Not reported | 2 (1.6) | 0 | 0 |  |  |  |
| **Race/Ethnicity (n [%])** |  |  |  |  |  |  |
| African American/Black | 5 (4.1) | 3 (2.7) | 5 (3.6) | 0.892 | 0.999 | 0.907 |
| Asian or Pacific Islander | 3 (2.4) | 4 (3.6) | 4 (2.9) |  |  |  |
| Hispanic | 7 (5.7) | 7 (6.3) | 8 (5.7) |  |  |  |
| White | 104 (84.6) | 96 (85.7) | 118 (84.3) |  |  |  |
| Multiple/Other | 4 (3.3) | 2 (1.8) | 5 (3.6) |  |  |  |
| **Employment type (n [%])** |  |  |  |  |  |  |
| Full-time | 48 (39.0) | 43 (38.4) | 37 (26.4) | 0.932 | 0.577 | 0.418 |
| Part-time | 12 (9.8) | 10 (8.9) | 16 (11.4) |  |  |  |
| Self-employed | 4 (3.3) | 5 (4.5) | 3 (2.1) |  |  |  |
| Homemaker | 15 (12.2) | 12 (10.7) | 24 (17.1) |  |  |  |
| Retired | 17 (13.8) | 18 (16.1) | 23 (16.4) |  |  |  |
| Unemployed | 14 (11.4) | 16 (14.3) | 20 (14.3) |  |  |  |
| Disabled | 8 (6.5) | 6 (5.4) | 13 (9.3) |  |  |  |
| Student | 3 (2.4) | 2 (1.8) | 3 (2.1) |  |  |  |
| [Decline to answer] | 2 (1.6) | 0 | 1 (0.7) |  |  |  |
| **Education (n [%])** |  |  |  |  |  |  |
| Less than high school | 2 (1.6) | 0 | 1 (0.7) | 0.139 | 0.925 | 0.096 |
| Completed some high school | 4 (3.3) | 4 (3.6) | 5 (3.6) |  |  |  |
| High school graduate or equivalent (e.g., GED) | 20 (16.3) | 14 (12.5) | 21 (15.0) |  |  |  |
| Completed some college, but no degree | 34 (27.6) | 25 (22.3) | 47 (33.6) |  |  |  |
| Associate’s degree | 18 (14.6) | 19 (17.0) | 22 (15.7) |  |  |  |
| College graduate (e.g., B.A., A.B., B.S.) | 25 (20.3) | 40 (35.7) | 26 (18.6) |  |  |  |
| Completed some graduate school, but no degree | 4 (3.3) | 4 (3.6) | 5 (3.6) |  |  |  |
| Completed graduate school (e.g., M.S., M.D., Ph.D.) | 15 (12.2) | 6 (5.4) | 13 (9.3) |  |  |  |
| [Decline to answer] | 1 (0.8) | 0 | 0 |  |  |  |
| **Residence (n [%])** |  |  |  |  |  |  |
| Urban/City | 26 (21.1) | 25 (22.3) | 37 (26.4) | 0.612 | 0.278 | 0.733 |
| Suburban | 71 (57.7) | 58 (51.8) | 67 (47.9) |  |  |  |
| Rural | 26 (21.1) | 29 (25.9) | 36 (25.7) |  |  |  |
| **Region (n [%])** |  |  |  |  |  |  |
| Northeast | 32 (26.0) | 24 (21.4) | 19 (13.6) | 0.099 | 0.001^a^ | 0.245 |
| South | 34 (27.6) | 19 (17.0) | 22 (15.7) |  |  |  |
| Midwest | 42 (34.1) | 48 (42.9) | 76 (54.3) |  |  |  |
| West | 15 (12.2) | 21 (18.8) | 23 (16.4) |  |  |  |
| **Household income (n [%])** |  |  |  |  |  |  |
| Less than $15,000 | 12 (9.8) | 7 (6.3) | 18 (12.9) | 0.738 | 0.029^a^ | 0.050 |
| $15,000 to $24,999 | 9 (7.3) | 11 (9.8) | 11 (7.9) |  |  |  |
| $25,000 to $34,999 | 8 (6.5) | 9 (8.0) | 16 (11.4) |  |  |  |
| $35,000 to $49,999 | 16 (13.0) | 15 (13.4) | 16 (11.4) |  |  |  |
| $50,000 to $74,999 | 18 (14.6) | 24 (21.4) | 38 (27.1) |  |  |  |
| $75,000 to $99,999 | 26 (21.1) | 19 (17.0) | 15 (10.7) |  |  |  |
| $100,000 to $124,999 | 12 (9.8) | 8 (7.1) | 15 (10.7) |  |  |  |
| $125,000 to $149,999 | 9 (7.3) | 4 (3.6) | 7 (5.0) |  |  |  |
| $150,000+ | 11 (8.9) | 12 (10.7) | 3 (2.1) |  |  |  |
| [Decline to answer] | 2 (1.6) | 3 (2.7) | 1 (0.7) |  |  |  |
| **Smoking Status (n [%])** |  |  |  |  |  |  |
| Never | 58 (47.2) | 56 (50.0) | 58 (41.4) | 0.877 | 0.534 | 0.805 |
| Used To | 32 (26.0) | 24 (21.4) | 32 (22.9) |  |  |  |
| Once a month or less | 2 (1.6) | 2 (1.8) | 7 (5.0) |  |  |  |
| 2-3 times per month | 2 (1.6) | 1 (0.9) | 3 (2.1) |  |  |  |
| Once a week | 1 (0.8) | 1 (0.9) | 1 (0.7) |  |  |  |
| 2-3 times per week | 0 | 2 (1.8) | 3 (2.1) |  |  |  |
| 4-6 times per week | 3 (2.4) | 3 (2.7) | 5 (3.6) |  |  |  |
| Daily | 25 (20.3) | 23 (20.5) | 31 (22.1) |  |  |  |
| **Alcohol Use (n [%])** |  |  |  |  |  |  |
| Never | 15 (12.2) | 9 (8.0) | 16 (11.4) | 0.249 | 0.458 | 0.182 |
| Used To | 21 (17.1) | 20 (17.9) | 28 (20.0) |  |  |  |
| Once a month or less | 25 (20.3) | 35 (31.3) | 28 (20.0) |  |  |  |
| 2-3 times per month | 18 (14.6) | 14 (12.5) | 21 (15.0) |  |  |  |
| Once a week | 18 (14.6) | 14 (12.5) | 22 (15.7) |  |  |  |
| 2-3 times per week | 18 (14.6) | 14 (12.5) | 10 (7.1) |  |  |  |
| 4-6 times per week | 6 (4.9) | 1 (0.9) | 7 (5.0) |  |  |  |
| Daily | 2 (1.6) | 5 (4.5) | 8 (5.7) |  |  |  |
| **Comorbidities (current) (n [%])** |  |  |  |  |  |  |
| AIDS/HIV | 0 | 1 (0.9) | 1 (0.7) | 0.477 | 1.000 | 1.000 |
| Asthma | 19 (15.4) | 24 (21.4) | 26 (18.6) | 0.236 | 0.502 | 0.572 |
| Cancer | 4 (3.3) | 9 (8.0) | 8 (5.7) | 0.153 | 0.389 | 0.465 |
| Cerebrovascular accident or transient ischemic attack | 0 | 2 (1.8) | 3 (2.1) | 0.226 | 0.250 | 1.000 |
| Chronic anxiety | 37 (30.1) | 32 (28.6) | 52 (37.1) | 0.800 | 0.227 | 0.152 |
| Chronic pulmonary disease (e.g., COPD, emphysema, chronic bronchitis) | 10 (8.1) | 13 (11.6) | 7 (5.0) | 0.370 | 0.303 | 0.054 |
| Congestive heart failure | 1 (0.8) | 3 (2.7) | 0 | 0.350 | 0.468 | 0.087 |
| Connective tissue disease (e.g., rheumatoid arthritis) | 2 (1.6) | 2 (1.8) | 4 (2.9) | 1.000 | 0.688 | 0.696 |
| Dementia | 0 | 1 (0.9) | 0 | 0.477 | – | 0.444 |
| Depression | 38 (30.9) | 42 (37.5) | 57 (40.7) | 0.335 | 0.098 | 0.604 |
| Diabetes (type 1) | 0 | 0 | 2 (1.4) | – | 0.500 | 0.504 |
| Diabetes (type 2) | 6 (4.9) | 7 (6.3) | 8 (5.7) | 0.646 | 0.763 | 0.858 |
| Hyperlipidemia | 5 (4.1) | 4 (3.6) | 4 (2.9) | 1.000 | 0.738 | 1.000 |
| Hypertension | 25 (20.3) | 24 (21.4) | 25 (17.9) | 0.835 | 0.611 | 0.477 |
| Irritable bowel syndrome | 8 (6.5) | 11 (9.8) | 22 (15.7) | 0.352 | 0.019^a^ | 0.168 |
| Liver disease | 2 (1.6) | 1 (0.9) | 3 (2.1) | 1.000 | 1.000 | 0.631 |
| Myocardial infarction | 1 (0.8) | 2 (1.8) | 1 (0.7) | 0.607 | 1.000 | 0.587 |
| Peptic ulcer disease | 0 | 0 | 2 (1.4) | – | 0.500 | 0.504 |
| Peripheral vascular disease | 0 | 2 (1.8) | 2 (1.4) | 0.226 | 0.500 | 1.000 |
| Sleep disorders (including sleep apnea) | 20 (16.3) | 22 (19.6) | 31 (22.1) | 0.499 | 0.229 | 0.629 |
| Ulcer | 2 (1.6) | 3 (2.7) | 5 (3.6) | 0.671 | 0.454 | 0.736 |
| **Comorbidities (ever diagnosed) (n [%])** |  |  |  |  |  |  |
| AIDS/HIV | 0 | 1 (0.9) | 1 (0.7) | 0.477 | 1.000 | 1.000 |
| Asthma | 21 (17.1) | 21 (18.8) | 26 (18.6) | 0.738 | 0.752 | 0.971 |
| Cancer | 5 (4.1) | 7 (6.3) | 11 (7.9) | 0.558 | 0.301 | 0.623 |
| Cerebrovascular accident or transient ischemic attack | 0 | 1 (0.9) | 2 (1.4) | 0.477 | 0.500 | 1.000 |
| Chronic anxiety | 35 (28.5) | 33 (29.5) | 51 (36.4) | 0.865 | 0.169 | 0.244 |
| Chronic pulmonary disease (e.g., COPD, emphysema, chronic bronchitis) | 9 (7.3) | 9 (8.0) | 7 (5.0) | 0.836 | 0.433 | 0.326 |
| Congestive heart failure | 2 (1.6) | 3 (2.7) | 1 (0.7) | 0.671 | 0.601 | 0.326 |
| Connective tissue disease (e.g., rheumatoid arthritis) | 3 (2.4) | 4 (3.6) | 4 (2.9) | 0.712 | 1.000 | 1.000 |
| Dementia | 0 | 0 | 0 | – | – | – |
| Depression | 44 (35.8) | 41 (36.6) | 58 (41.4) | 0.894 | 0.348 | 0.436 |
| Diabetes (type 1) | 1 (0.8) | 0 | 2 (1.4) | 1.000 | 1.000 | 0.504 |
| Diabetes (type 2) | 7 (5.7) | 6 (5.4) | 10 (7.1) | 0.911 | 0.633 | 0.564 |
| Hyperlipidemia | 2 (1.6) | 5 (4.5) | 3 (2.1) | 0.263 | 1.000 | 0.472 |
| Hypertension | 26 (21.1) | 25 (22.3) | 28 (20.0) | 0.826 | 0.820 | 0.653 |
| Irritable bowel syndrome | 10 (8.1) | 15 (13.4) | 22 (15.7) | 0.191 | 0.061 | 0.605 |
| Liver disease | 3 (2.4) | 2 (1.8) | 4 (2.9) | 1.000 | 1.000 | 0.696 |
| Myocardial infarction | 1 (0.8) | 1 (0.9) | 1 (0.7) | 1.000 | 1.000 | 1.000 |
| Peptic ulcer disease | 0 | 1 (0.9) | 1 (0.7) | 0.477 | 1.000 | 1.000 |
| Peripheral vascular disease | 0 | 2 (1.8) | 1 (0.7) | 0.226 | 1.000 | 0.587 |
| Sleep disorders (including sleep apnea) | 21 (17.1) | 21 (18.8) | 34 (24.3) | 0.738 | 0.151 | 0.290 |
| Ulcer | 4 (3.3) | 5 (4.5) | 4 (2.9) | 0.740 | 1.000 | 0.516 |
| **Resources Used in Past 12 Months** |  |  |  |  |  |  |
| Primary care physician | 111 (90.2) | 104 (92.9) | 128 (91.4) | 0.473 | 0.739 | 0.677 |
| Specialist | 57 (46.3) | 43 (38.4) | 67 (47.9) | 0.218 | 0.806 | 0.132 |
| Urgent care facility | 50 (40.7) | 31 (27.7) | 54 (38.6) | 0.037^a^ | 0.731 | 0.069 |
| Emergency room visit | 28 (22.8) | 26 (23.2) | 47 (33.6) | 0.935 | 0.053 | 0.072 |
| Hospital (admitted/hospitalized) | 9 (7.3) | 10 (8.9) | 17 (12.1) | 0.651 | 0.191 | 0.412 |
| Other | 4 (3.3) | 7 (6.3) | 6 (4.3) | 0.359 | 0.755 | 0.572 |

^a^ Statistically significant difference (p < 0.05). Acquired Immunodeficiency Syndrome (AIDS). Antibiotics (AB). Chronic Obstructive Pulmonary Disease (COPD). Human Immunodeficiency Virus (HIV). General Educational Development (GED). Uncomplicated urinary tract infection (uUTI).

## Treatment satisfaction

Treatment satisfaction composite scores (mean [standard deviation]) for global satisfaction, effectiveness, and convenience were 76.5 (20.8), 70.8 (21.2), and 82.8 (17.3), respectively (S4 Table). Participants with non-recurrent uUTI had significantly higher mean effectiveness scores than those with recurrent uUTI (73.8 vs. 66.5; p = 0.002; S4 Table). Mean effectiveness scores were also higher for participants who received only 1 antibiotic for their most recent uUTI vs. those who received ≥ 3 (72.7 vs. 64.5; p = 0.017; S5 Table). Mean convenience scores were higher for participants who received just 1 antibiotic vs. those who received 2 (84.8 vs. 79.0; p = 0.008; S5 Table); and for participants who received just 1 appropriate antibiotic vs. those who received multiple (85.6 vs. 79.4; p = 0.004; S5 Table).

**S4 Table. Treatment satisfaction overall and stratified by uUTI recurrence.**

| **Treatment satisfaction, mean (SD)** | **Overall  (n = 375)** | **Recurrent  (n = 163)** | **Non-recurrent  (n = 212)** | **Recurrent vs. non-recurrent  (p-value)** |
| --- | --- | --- | --- | --- |
| Global Satisfaction Score | 76.5 (20.8) | 74.3 (20.4) | 77.9 (21.1) | 0.126 |
| Effectiveness Score | 70.8 (21.2) | 66.5 (21.2) | 73.8 (20.7) | 0.002^a^ |
| Convenience Score | 82.8 (17.3) | 81.1 (18.4) | 84.0 (16.6) | 0.139 |

^a^ Statistically significant (p < 0.05).
Standard deviation (SD). Uncomplicated urinary tract infection (uUTI).

**S5 Table. Treatment satisfaction stratified by number of AB used and appropriateness of therapy.**

| **Treatment satisfaction,  mean (SD)** | **1 AB (n = 235)** | **2 AB (n = 88)** | **≥ 3 AB (n = 52)** | **P-value for comparison** | | |
| --- | --- | --- | --- | --- | --- | --- |
|  |  |  |  | **1 vs. 2 AB** | **1 vs. ≥ 3 AB** | **2 vs. ≥ 3 AB** |
| Global Satisfaction Score | 77.8 (20.6) | 75.2 (20.7) | 72.8 (21.8) | 0.349 | 0.142 | 0.542 |
| Effectiveness Score | 72.7 (20.8) | 69.7 (21.2) | 64.5 (21.9) | 0.278 | 0.017^a^ | 0.199 |
| Convenience Score | 84.8 (16.9) | 79.0 (15.7) | 80.1 (20.5) | 0.008^a^ | 0.100 | 0.741 |
|  | **1 appropriate AB  (n = 123)** | **1 inappropriate AB  (n = 112)** | **Multiple ABs  (n = 140)** | **Appropriate vs. inappropriate** | **Appropriate vs. multiple** | **Inappropriate vs. multiple** |
| Global Satisfaction Score | 77.0 (21.8) | 78.8 (18.9) | 74.3 (21.1) | 0.531 | 0.319 | 0.110 |
| Effectiveness Score | 72.4 (21.4) | 73.0 (20.0) | 67.7 (21.5) | 0.853 | 0.085 | 0.073 |
| Convenience Score | 85.6 (15.7) | 83.8 (18.6) | 79.4 (17.6) | 0.446 | 0.004^a^ | 0.084 |

^a^ Statistically significant (p < 0.05).
Antibiotic (AB). Standard deviation (SD).

# Reference

1. Bharmal M, Payne K, Atkinson MJ, Desrosiers M-P, Morisky DE, Gemmen E. Validation of an abbreviated Treatment Satisfaction Questionnaire for Medication (TSQM-9) among patients on antihypertensive medications. Health Qual Life Outcomes. 2009;7:36.
